# Power asymmetries in global governance for health: a conceptual framework for analyzing the political-economic determinants of health inequities

**DOI:** 10.1186/s12992-019-0516-4

**Published:** 2019-11-28

**Authors:** Alexander Kentikelenis, Connor Rochford

**Affiliations:** 1 Department of Social and Political Sciences, Bocconi University, Milan, Italy; 2 Centre for Global Health Inequalities Research (CHAIN), Norwegian University of Science and Technology, Trondheim, Norway; 3 Blavatnik School of Government, University of Oxford, Oxford, UK

**Keywords:** Global governance for health, Health inequality, Political economy of health, Power asymmetries

## Abstract

**Background:**

Recent scholarship has increasingly identified global power asymmetries as the root cause of health inequities. This article examines how such asymmetries manifest in global governance for health, and how this impacts health outcomes.

**Results:**

We focus on the political-economic determinants of global health inequities, and how these determinants operate at different levels of social action (micro, meso, and macro) through distinct but interacting mechanisms. To clarify how these mechanisms operate, we develop an integrative framework for examining the links between global neoliberalism—the currently dominant policy paradigm premised on advancing the reach of markets and promoting ever-growing international economic integration—and global health inequities, and show how these mechanisms have macro–macro, macro–meso–macro, and macro–micro–macro manifestations.

**Conclusions:**

Our approach enables the design of theoretically-nuanced empirical strategies to document the multiple ways in which the political economy entrenches or, alternatively, might ameliorate global health inequities.

## Background

Global health inequities increasingly result from the activities of transnational actors with varying interests, degrees of power, and ways of operating [1–4]. Analyses of the determinants of such inequities must be able to identify and unpack the political-economic processes underpinning these activities: from micro-level interventions to combat disease, to organizational decision-making over health policy, and to global norm-making about access to healthcare and medicines. This task is rendered difficult by the rapid expansion of actors that impact global health. Ten years ago there were 175 initiatives, funds, agencies, and donors with a primary remit on health, whereas recent studies indicate there are now some 203 such actors [5, 6]. However, even that net is cast too narrowly: a range of new or traditional actors are (re)shaping the global health field directly (as through interventions) or indirectly (as through the follow-on effects on health resulting from economic or political decisions). Understanding how these actors make decisions and seek to engage in and influence policy is imperative for generating fine-grained explanations of the political-economic origins of health inequities [2, 7, 8].

Reflecting on these transformations, in 2015 the Commission on Global Governance for Health—sponsored by *The Lancet* and the University of Oslo—set out a research and policy agenda that emphasized politics as the root cause of health inequities, whether within or between countries [1]. Central here is the concept of *power asymmetries*: the unequal distribution of different types of resources (like money, knowledge or political authority) across the array of actors involved in governance arrangements affecting health. By extension, then, tackling global health inequities requires action on their political-economic origins.

In this article, we examine how such asymmetries manifest in global governance for health—and, in turn, how this impacts health outcomes. To trace power asymmetries, we focus on the political-economic determinants of global health inequities, and how these determinants operate at micro-, meso- and macro-levels of social action through distinct but interacting mechanisms. To clarify how these mechanisms operate, we develop an integrative framework for investigating the links between global neoliberalism—the currently dominant policy paradigm premised on advancing the reach of markets and promoting ever-growing international economic integration—and global health inequities, showing how these mechanisms have macro–macro, macro–meso–macro, and macro–micro–macro manifestations. This approach enables the design of theoretically-nuanced empirical strategies to document the many ways in which the political economy entrenches, or, alternatively, might ameliorate global health inequities.

## Methods

Our study draws on academic literature and other publicly available sources (such as reports by international or non-governmental organizations) to map out how power asymmetries manifest in global governance for health, and how this affects global health inequities. Our aim is not to produce a systematic review of scholarly work on these topics, as that has been undertaken by numerous other studies [9–13]. Instead, we opt for a narrative synthesis that utilizes conceptual advances in the social sciences to shed additional light on public health outcomes: starting from theoretical approaches to power and how it manifests at different levels of analysis, we then posit plausible links between the variables of interest. Subsequently, we populate that framework with empirical evidence in literature identified through Web of Science and Google Scholar searches. This exploratory and syncretic approach is well-suited to our main objectives of advancing theory frontiers, examining pathways linking the variables of interest, and exploring promising avenues for future research [14].

## Results and discussion

### Global governance for health in flux

It has become commonplace to note that the governance arrangements impacting health encompass an increasing number of actors that are bound together through complex linkages [2, 6, 7, 15–20]. “Traditional” actors in global health—states and international organizations like the World Health Organization (WHO)—remain centrally important, but their activities are complemented by a host of newer actors: non-governmental organizations (NGOs), philanthropic foundations (most notably, the Gates Foundation), public–private partnerships like Gavi and the Global Fund to Fight AIDS, Tuberculosis and Malaria, pharmaceutical companies, and prominent centers of knowledge production have all reshaped how decisions are made and what evidence is to be taken into account. In addition, the global health community has recognized that also actors without an explicit remit on health—ranging from major multinational companies to intergovernmental organizations structuring international economic relations—also directly impact global health. This expansion in the number of actors and issues has led to growing multipolarity and fragmentation in the field of global health [21].

The many actors involved interact in a range of politically-charged terrains: from local-level health interventions that encompass multiple interested parties, to national or regional policy-making, and even to global processes that shape health-impacting regulations or the broader norms underpinning policy actions. For example, interventions to combat HIV transmission in severely affected low-income countries are often administered jointly by local governmental and non-governmental organizations, but are funded by the Global Fund (a global public–private partnership with no footprint in recipient countries) and benefit from technical assistance from the WHO and donor states (intergovernmental and third-state actors) [20]. Further, the development of global norms—“behavioral prescriptions that are accepted by subjects as legitimate and authoritative” [22–24]—on improving access to affordable antiretroviral medications has rested on an iterative process whereby developing countries have exploited flexible arrangements within the intellectual property rights regime to advance their own interpretations of the obligations of states and pharmaceutical companies [25]. As these examples indicate, global governance for health manifests across a range of terrains where actors (local, national, global) interact in shaping the outcomes that influence population health.

This cursory discussion indicates that global governance for health arrangements are indeed affected by power asymmetries: there is no level playing field among equal actors, but an imbalanced decision-making apparatus where different actors command varying degrees of political, economic, symbolic, or epistemic power. These topics have received extensive recognition in earlier studies, but there are two major limitations with the prevailing conceptualization and analysis. The first concerns viewing actions as if they occurred in independent spheres, rather than in interconnected terrains of activity. The second is the overwhelming emphasis on actors and their actions as the primary units of analysis. This approach commonly focuses on the behavior of individuals or organizations to explain health outcomes, avoiding broader institutional explanations [26–28]. Drawing on recent advances in sociological theory [29], we advocate for an approach that includes recognition of both individual agency and institutional structures, while also factoring in how organizations in their various manifestations connect the micro-actions and macro-structures. Employing such a layered approach will help in developing more complete accounts of the determinants of health inequities.

### Studying power and power asymmetries in global governance for health

Increased complexity and multipolarity in global governance for health can obscure how power manifests and how power asymmetries entrench health inequalities. To shed light on these social processes, we turn to social-scientific scholarship: first, to examine how differing conceptualizations of power can have analytical traction for approaching issues in global governance for health, and subsequently to outline the constellation of power asymmetries in this field.

Of course, the concept of power has been foundational for social inquiry. Max Weber famously understood power as involving the ability of an actor or group of actors “to realize their own will in a social action even against the resistance of others who are participating in the action” [30]. While this definition is seems intuitively correct, and focuses explicitly on power exercised in the course of making decisions, it can mask the fact that non-decisions may be equally important: “some issues are organized into politics while others are organized out” [31]–and this is itself a process rife with power asymmetries. For instance, the pharmaceutical industry is able to achieve “regulatory capture” of key public agencies and use financial links to politicians to prevent unfavorable legislation from emerging in the first place [32]. A third clarification of the concept of power adds recognition of the institutional environment that sets the broad parameters defining which topics may be subject to decision-making or non-decision-making [33]. In other words, power is exercised not only by impacting decisions or preventing issues from entering the arena of decision-making, but also by shaping the desires, preferences, and actions of less-powerful societal groups. For example, the rise of large philanthropic foundations in global health and development has also been associated with emerging ideas about who is found deserving of charity on the basis of the views and interests of philanthropists [34], and not on the basis of principles of justice and redistribution that have emerged through collective processes. This, in turn, can have important implications for how less-powerful individuals or organizations position themselves in order to be deemed worthy of assistance.

This discussion draws attention to the range of social relations that can carry the imprint of power. The specific manifestations of power relationships depend on the nature of the power resources—e.g., political, economic, epistemic, or symbolic—held by various actors [35]. This is not to say that holding these resources is mutually exclusive. Economic and political power often overlap, as evidenced by the ability of economic elites to skew politics and public discourse towards their own goals [36]. Or epistemic power can be political, as powerful actors can strive to advance their own epistemic preferences [37]—this is particularly important for policy areas, like economics, where there is limited consensus about appropriate policy action.

Notably, the power of political authorities—primarily states, but also important intergovernmental organizations due to the “public-ness of their purpose” [38]—may take various forms. It can be coercive, exercised with scant regard for including other actors in the decision-making process. For example, scholarship in international relations has documented how powerful countries use coercion—whether acting by themselves or through international organizations—to induce policy change elsewhere in the world [39–42]. However, political authorities usually do not rely on outright coercion, but instead utilize rules and regulations—and an expansive bureaucratic apparatus—to ensure the enforcement of policies across a given territory [43]. Thus, political power comes into much greater contact with other forms of power, as groups beyond political actors in the strict sense are also involved in the inception, design, and implementation of policies [43]. For instance, multilateral negotiations over intellectual property rights and access to medicines have included states and pharmaceutical companies, but also national and global health activists and NGOs [44].

Power resources beyond political power have a profound impact on decision-making, with follow-on implications for health outcomes. Economic power is a core resource that private actors have at their disposal, and they exercise it accordingly. This occurs in many ways within borders—for example, through regulatory capture, funding sympathetic politicians, or engaging in public relations campaigns—but also has important international manifestations. In 2010, tobacco giant Philip Morris sued (ultimately, unsuccessfully) Uruguay for damages when the latter introduced legislation on cigarette packaging [45]—a legal strategy pursued by tobacco companies elsewhere as well. In addition, epistemic power is a key input in broader policy-making that impacts health inequalities, as universities and research organizations collect data and conduct independent analyses of the determinants of health and the impact of policy interventions [46]. Finally, symbolic power—power over generating a “legitimate vision of the world” [47]—is also crucially important for agenda setting and political struggles. For example, the WHO is the key organization with the symbolic authority to issue influential declarations on health issues, which in turn places it at the center of power struggles involving states, businesses, experts, and NGOs [48].

Given these multiple dimensions and types of power, how do power asymmetries manifest in global governance for health? First, there can be considerable within-actor heterogeneity. For example, struggles over health policy within countries have to confront the fact that the fiscal authorities may have different priorities than investing in health, and may constrain available financial resources; on the other hand, securing buy-in from finance ministries early on in the development of health interventions can secure sustainable long-term support for budding initiatives [49]. Or, with wealthier countries, their global health-related activities may be subject to tensions between humanitarian objectives of health promotion, and foreign policy or national security objectives—and such different types of concerns can have important implications for the outcomes of health interventions [50].

Second, power asymmetries are clearly manifested in relations between actors: between different states, between states and businesses, between intergovernmental and non-governmental organizations, or between businesses and experts. Two illustrations will suffice here. In the 1970s, developing countries sought to get WHO policy-making re-oriented towards greater recognition of the health impact of the social conditions in which people are born, grow, work, and live; however, powerful interests in Western countries blocked such a redefinition of the WHO’s remit beyond the narrower focus on the proximate, biomedical determinants of health [48, 51]. Or, as an illustration of power asymmetries between different types of actors, major philanthropic foundations—because of the magnitude of funds they command—have the ability to (re)shape the policy agendas, research priorities, and strategies of NGOs operating in global health [52, 53]. For instance, as one of the largest funders in global health, the Bill and Melinda Gates Foundation has been able to steer research and interventions in areas that the Foundation itself considers as top priorities, although these are not always the most important drivers of the disease burden within countries [54]. Or, to take a different instance, the Gates Foundation partnered with the Coca-Cola Company (in which the Foundation is a major shareholder) to develop new business opportunities for farmers who sell fruit to the Company in Uganda and Kenya [52]. According to the official narrative, this partnership would increase farmers’ incomes and also help fight hunger [55]. However, the activities of the sugary drinks industry, including the Coca-Cola Company, have well-established links to unhealthy lifestyles and the incidence of obesity and other health problems [56–59]. In short, these are cases where wealthy philanthropic foundations may promote ideas or policies that can have adverse—albeit probably unintended—consequences for health equity.

Comprehensively modelling these power asymmetries is inevitably a contextual issue: how these asymmetries manifest—and what types of power resources are most pertinent for actors—depends on the specific public health issue under investigation. Nonetheless, the empirical exercise of tracing how power asymmetries impact global health inequities can be organized in a comprehensive analytical framework that enables mapping complex links across different levels of policy action. That is the task of the remainder of this article.

### Bringing in multiple levels of analysis

Given unequal and entrenched power relations, how do power asymmetries manifest in different settings of global governance for health? We distinguish between three levels of analysis [29]. First, the institutional level—also referred to as the “macro” level—is where “complexes of routines, rules, roles, and meanings” [29] reside, simultaneously shaping and shaped by social action. In other words, institutions have regulative, normative and cognitive dimensions that guide the behavior of organizations and individuals [60], and are themselves products of past power struggles. Institutional, macro-level analysis is focused on elaborating on how institutions came to be in the first place, how they change, and how they structure social and policy environments.

Second, the “meso” level of analysis is also a key locus where power asymmetries manifest. Here we find organizations operating within an institutional environment. That is, organizations come to embody different aspects of institutions, carrying them forward in their everyday activities [61, 62]. For example, the World Bank and the International Monetary Fund (IMF)—the premier global economic governance organizations—came to embody the neoliberal policy orthodoxy from the 1980s onwards, which in turn produced policy prescriptions that favored competition, efficiency, and shrinking the public sector [63–66]. Its advocacy of user fees for access to healthcare provides a case in point: applying neoliberal orthodoxy to health-policy practice, World Bank economists evangelized the introduction of user fees as a way of rationing and delivering better services—neglecting or ignoring much evidence that did not correspond to this rationality [67, 68]. However, as noted below, these are not monolithic organizations, nor do they necessarily advocate identical policy prescriptions [69, 70].

Third, power asymmetries also manifest at the level of individuals, or the “micro” level. Some individuals have more power resources—e.g., money, networks, or positions of authority—than others to advance their values and interests. However, it is important not to view individuals as all-powerful free-floating actors. Rather, they are best seen as “enactors” of the dominant institutional orders: individuals “derive their identities and interests from some perceived natural order” [71], which is imprinted on them by virtue of their socialization, whether at home or through their training [37, 72, 73]. Individuals act according to certain cultural models or scripts, which they interpret, fine-tune, and expand [61]. For example, “global health” became a degree specialization in many universities and medical schools in the 2000s, and towards the end of the decade, the Gates and Rockefeller foundations funded attempts to standardize the curricula of global health programs in order to ensure that future professionals would have comparable training and skills. However, this entailed framing *one* set of issues as relevant to developing countries, potentially narrowing down the intellectual and policy horizons of an emerging generation of health professionals—for example, by promoting certain “best practices” of health system organization, or by directing attention away from structural causes of ill health [74, 75]. That is not to say that individuals never have space or opportunity to transcend the dominant institutional order or resist its key tenets: even seemingly weak actors can sometimes escape from dominant institutional logics [76]. Rather, the emphasis on the boundedness of agency is intended to highlight the real constraints—material or otherwise—on human action that restrict the ability to lead a healthy life.

Further, individual choices carry imprints of the dominant institutional order. For example, the availability of decent jobs, eligibility for health services, or access to healthy food all determine individual health choices, even though they are the products of structural forces often far removed from people’s everyday experiences. A case in point concerns individual choices for accessing healthcare: in the context of the highly exclusionary and expensive US health policy model (a macro-level force), some individuals turn to crowdfunding as the ultimate means to access healthcare, thereby commodifying and mediatizing their experiences of illness [77]. In short, institutional characteristics, often supported by powerful private actors who seek to further their interests, result in resistance to change, thereby hampering the ability of individuals to make healthy choices [57, 78].

These three levels of analysis can help to cast light on how power asymmetries manifest in practice, and the examples presented have been drawn from different areas of social action that impact health. However, the analysis thus far has not attempted to indicate how these levels can be brought together into a unified explanatory framework for mapping the mechanisms that link the observed phenomena. To this we now turn.

### Global neoliberalism and health inequity: an integrative framework of analysis

To demonstrate the utility of approaching power asymmetries in global governance for health from a “levels of analysis” perspective, we present an integrative framework that enables tracing the relevant pathways of influence. Our explanandum is the prevalence of health inequities, understood as a systemic feature of the contemporary world. Such inequities are rooted in individual-level health behaviors, but they are also impacted by a range of additional processes, as discussed below.

We trace the determinants of health inequities that relate to global neoliberalism. The latter refers to the particular political-economic paradigm that emerged in the final quarter of the twentieth century, promoting free markets and individual rights as organizing principles of collective life [79–83]. According to this paradigm, deregulating economic activity from purportedly harmful and counterproductive government regulations was seen as the best policy prescription for countries around the world. This paradigm translated into the growing liberalization of trade and capital flows, reduction of governmental regulations over economic activity, and the privatization of public entities and natural resources.

Neoliberalism initially found fertile ground in the USA, the UK, and some Western-backed authoritarian regimes. Then, from the mid-1980s onwards, it spread across the world through various social processes, including the authority of experts, the imperative for countries to remain “competitive” under conditions of rapidly growing international economic integration, and coercion by international organizations like the IMF and the World Bank [39, 40, 84–87]. Consequently, we focus here on *global* neoliberalism as the dominant institutional order of the contemporary world. This is an order that was instituted and is maintained through power asymmetries (e.g., by Western countries and Western-dominated organizations, or by powerful business groups) [41, 63, 64], and whose outcome has been to further entrench power asymmetries (e.g., as seen in the concentration of income and wealth in the hands of fewer and fewer people, primarily—but not exclusively—in the West) [88].

**Fig. 1:**
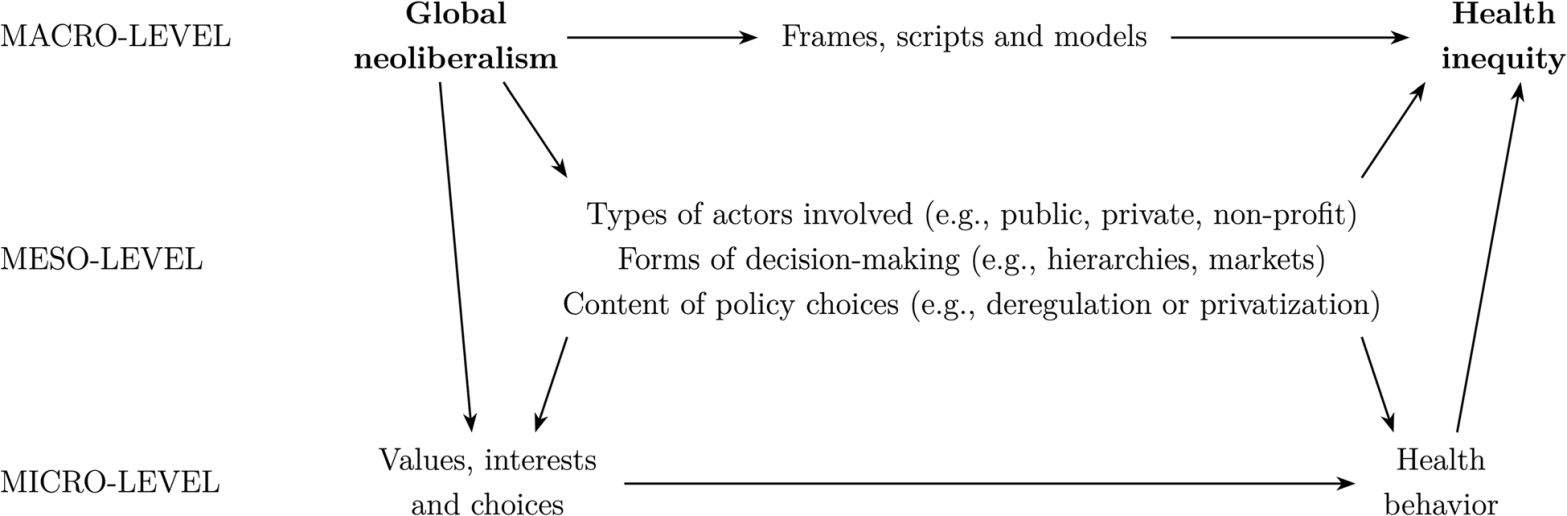
Multiple levels of analysis and the origins of health inequity. *Source*: Authors, drawing on Jepperson and Meyer [29]

Figure 1 maps the multiple pathways through which global neoliberalism affects health inequities. We understand such inequities in broad terms, as referring to health risks that exhibit a social gradient (i.e., differentially affecting certain strata of the population). Ultimately, these risks become reflected in aggregate population health data, which can obscure the aforementioned social gradients. For example, in the European context, aggregate data on mental health mask the fact that a person with low socio-economic status (approximated by low levels of education) is three times more likely to report depression than someone who has completed tertiary education [89].

First, one key pathway runs through macro–micro–macro processes: emanating from the dominant institutional order, neoliberal frames become internalized by individuals around the world, and are in turn reflected in their ideas, values, and choices. Ideas and values are important, as they are tools that people employ to make sense of the world. For example, neoliberalism’s focus on the individual can turn collective policy problems into questions of individual worth and merit, which in turn can impact well-being (e.g., mental health status) [90, 91]. Micro-level processes are also directly impacted by the prevailing value systems: for instance, ordinary people can vote for political parties that promise to reduce purportedly extravagant social provisions and welfare entitlements, and policy elites may wish to advance neoliberal values when designing public policies or engaging in global negotiations over health-related issues [92–95]. More generally, neoliberalism creeps into individual behaviors regarding lifestyle, nutrition, and healthcare. For example, unhealthy diets result partly from the insufficient regulation of the food and beverage industry, and reduced ability to purchase healthy food due to shrinking real incomes [83, 96, 97].

Second, health inequities are substantially shaped by meso-level processes that themselves carry imprints of global neoliberalism. This meso-level includes various types of actors who employ distinct forms of decision-making to develop an array of policies. As to the terms of types of actors involved, the neoliberal era has promoted the increased prominence of the private sector in health, with a shift away from purely public solutions [98]. Also the forms of organizational decision-making reflect neoliberal approaches (e.g., introducing market-based approaches within public or non-profit organizations), and the logics of efficiency, profit-maximization, and managerialism have impacted organizational outputs: welfare policies are increasingly becoming marketized, health systems are facing pressures for privatization, and the regulation of health hazards (like tobacco and sugary or alcoholic beverages) is often blocked or dismantled. For instance, governments across Europe have introduced or increased user fees or co-payments for healthcare [89, 99, 100]; and many developing countries have unsuccessfully sought to introduce stricter tobacco regulations [57, 101, 102]. Similarly, corporations have used their financial resources to bankroll ostensibly neutral information campaigns that in fact serve to advance business aims, as with Coca Cola’s establishing the Global Energy Balance Network that promoted biased advice on how to combat obesity [56].

The role of the trade regime merits special mention. The gradual displacement of the World Trade Organization (WTO)—where groups of weaker countries had institutionalized possibilities to express dissent or promote their preferences—by regional and bilateral free trade agreements has exacerbated existing power asymmetries [103]. In particular, the rise of investor–state dispute settlement mechanisms within these agreements can expose countries to lengthy and costly legal battles when seeking to introduce measures that protect health. To be sure, trade agreements can also potentially improve health (for example, by increasing incomes of some groups, in turn enabling them to lead healthier lives). However, the possible benefits must be weighed against the potential health-damaging effects, including the restriction of policy space available for implementing measures to combat health inequalities.

Third, turning to macro–macro links, policy-making institutions and the ideas and practices associated with neoliberalism reinforce medical-individualist models of health and strengthen actors with material interests opposed to policies that would increase health equity, like companies wanting to avoid paying additional taxes [104]. These links legitimate only a limited set of policy actions (those that favor “individual responsibility” and minimal regulation or taxation of big business) and construct very narrow horizons of possibility for the pursuit of redistributive policies [91, 105]. Further, global neoliberalism can frame social inequalities—including those in health—in ways that make them difficult to address through interventions, abrogating any sense of political responsibility from policymakers and reducing the scope for collective-action strategies to address the drivers of increased inequalities. Global neoliberalism has legitimated a set of policy models that exhibit little consideration for inequality issues (although they can be more attuned to poverty issues [106]), and can thereby entrench health inequities around the world. That need not preclude the possibility of altering the prevalence of health inequities through intervention (a meso-level process): we merely point out that macro–macro links may make the possibility of intervention inconceivable for policy-makers or the general public.

### Limitations

The conceptual framework proposed here seeks to clarify how power asymmetries manifest at different levels of analysis, thereby reducing and structuring the complexity of the underlying processes. However, this approach is not without theoretical or empirical limitations. First, processes operating at different levels of analysis may be more important at different timepoints or for distinct explanatory purposes. For example, analyses aimed at explaining how multinational pharmaceutical companies seek to shape the global intellectual property rights regime need not delve into overly micro-mechanisms, but instead focus primarily on the relevant meso-level interactions. In any case, identifying the levels of analysis most pertinent to an analytical aim “should be an empirical rather than doctrinal matter” [29].

Second, in discussing the activities of “neoliberal” organizations, we have not meant to suggest that these are monolithic institutions, with no space for promoting policies that might yield a reduction in health inequities. For example, the World Bank and the WTO have certainly advanced a version of globalization that restricts the policy space for states and augments markets (with follow-on implications for health), but they also have engaged in or enabled activities—like the WTO’s agreeing to some exceptions in the protection of intellectual property rights that facilitated improved access to HIV medication [25]—that could improve population health. Or, to return to the Gates Foundation discussed above, one strand of its activities may advance the interests of major multinationals with possible negative effects on health equity, whereas another strand may actually contribute to decreasing social gradients in health (e.g., by limiting the spread of infectious diseases). In other words, analyses of organizational, meso-level processes that affect public health need to be attuned to the possible heterogeneity in the ways in which different organizations affect health equity in different contexts.

Third, the discussion of the political-economic mechanisms underpinning global health inequities is inevitably incomplete: there are myriad such pathways, and undertaking the kind of integrative analysis proposed above in any single empirical study is probably impossible. Nonetheless, approaching the determinants of such inequities from a “levels of analysis” perspective offers a conceptual apparatus to aid empirical enquiries into related questions. Measuring the impact of different mechanisms, identifying new ones, and clarifying their complex interactions can further our understanding of how political-economic choices affect individual health—and, by extension, reveal aggregate trends in global health inequities. Indeed, the variety of processes at different levels of analysis are indicative of the task at hand for future empirical research.

## Conclusions

This article has proposed a novel theoretical framework for approaching the determinants of global health inequities. To model these determinants comprehensively, analyses must pay attention to processes operating at various levels of analysis: from individual-level behaviors, to organizational decision-making, and right up to the institutional structures that frame policy thinking and individual actions. We have demonstrated the utility of this approach by analyzing the ways in which neoliberalism impacts global health inequities.

Although we have identified global neoliberalism as a dominant institutional feature of the contemporary world system, it is not the only such systemic factor that impacts health inequities. Alternative institutional frames—like human rights-based normative orders, and global egalitarian and emancipatory social norms—may open up pathways for reducing health inequities. Recent calls from the UN Human Rights Council for conducting health impact assessments at the onset of the design of public policies can be heeded [107] in order to clarify potential trade-offs and generate public awareness.

Future research can take on this theoretical and methodological agenda to advance a more comprehensive approach towards modelling the determinants of health inequities. Not all pathways may be amenable to perfect identification strategies, and no empirical study can simultaneously capture all the complex causal processes at play. Nonetheless, a conceptual appreciation of multiple and multi-level determinants will help highlight the political economy behind their emergence or persistence, and the ways in which they can be affected by global governance for health. For instance, analyses of inadequate access to medicines for some social groups should not examine only individual behaviors that lead to this, but also the meso-level, organizational processes of the WTO and investment treaties that determine the legal framework underpinning intellectual property rights. Also such analysis should be complemented with recognition of the macro-level institutional structures that led to the very emergence of this trading regime. At minimum, future work can shed light on the political-economic arrangements within which health inequities are situated. Otherwise, debates over how to understand and limit such inequalities will inevitably be incomplete.

## Data Availability

Not applicable.

## Abbreviations

IMF: International Monetary Fund
NGOs: Non-governmental organizations
WHO: World Health Organization
WTO: World Trade Organization
